# Head sweat rate prediction for thermal comfort assessment of bicycle helmets

**DOI:** 10.1186/2046-7648-4-S1-A85

**Published:** 2015-09-14

**Authors:** Peter Bröde, Guido De Bruyne, Jean-Marie Aerts, Tiago S Mayor, Dusan Fiala

**Affiliations:** 1 Leibniz Research Centre for Working Environment and Human Factors (IfADo), Dortmund, Germany; 2 University of Antwerp, Belgium; 3 KU Leuven, Belgium; 4 EMPA, St. Gallen, Switzerland; 5 Ergonsim, Marxzell, Germany; 6 HOPE-Helmet OPtimization in Europe, EU COST Action TU1101 Working Group 4 with the participation of JM Aerts, SA Annaheim, CP Bogerd, P Bröde, G De Bruyne, AD Flouris, K Kuklane, TS Mayor, RM Rossi (http://www.bicycle-helmets.eu

## Introduction

Only 1% to 40% of adult cyclists in European countries make use of bicycle helmets. This may be partly attributable to impaired thermal comfort associated with helmet use, which can lead to locally accumulated sweat and increased skin wettedness [1]. Local sweat rates (LSR) can be modelled by sudomotor sensitivities (SUD) relating the change in LSR to the change in body core temperature (TC) [2], or by the ratio of LSR to gross sweat rate (GSR) of the whole body [3]. Coupling those local models with models of thermoregulation predicting TC and GSR provides a framework for predicting head sweat rates in response to the characteristics of the thermal environment, clothing, level of activity and exposure time. This paper studies the influence of different local and whole-body models on predictive accuracy.

## Methods

We identified six different local models for the head region relying on SUD [2-4] and three models using LSR to GSR ratios [3]. These were linked with the models "Predictive Heat Strain" (PHS) [5], the multi-node UTCI-Fiala model of thermoregulation [6] and the still more complex "Fiala thermal Physiology and Comfort" (FPC) model [7]. We compared the prognoses to published means with 95% CI of LSR measured at the frontal and lateral head during bicycle ergometer trials with varying air temperatures (16 - 28 °C), air velocities (0.1 - 3 m·s^-1^) and activity levels (power output 50 - 150 W) [8,9].

## Results

Models based on SUD overestimated frontal head LSR, whereas for two SUD models predicted lateral LSR were within the experimental 95% CI, but with absolute percentage error between 3% and 36%. GSR based predictions were lower than LSR from SUD and covered better the 95% CI for the forehead, but again error ranged from 8% - 30%. Though PHS gave less accurate predictions than UTCI-Fiala and FPC, the local model determined overall performance, which was best for one GSR and two SUD models [3,4].

## Discussion

The poor prediction of forehead LSR, especially by models using SUD, may be improved by considering the modifying effect of local convective cooling [10], which is relevant to cycling.

## Conclusion

Eventually, integrating head LSR prediction with the calculation of skin wettedness in relation to evaporative resistance will provide assessment criteria for the thermal comfort of bicycle helmets.
